# Prevalence and factors associated with female genital mutilation among women of reproductive age in the Bawku municipality and Pusiga District of northern Ghana

**DOI:** 10.1186/s12905-018-0643-8

**Published:** 2018-09-18

**Authors:** Evelyn Sakeah, Cornelius Debpuur, Abraham Rexford Oduro, Paul Welaga, Raymond Aborigo, James Kotuah Sakeah, Cheryl A. Moyer

**Affiliations:** 1 Navrongo Health Research Centre, Post Office Box 114, Navrongo, Upper East Region Ghana; 2 Department of Medicine, University of Calgary, Calgary, AB Canada; 3 University of Michigan Medical School, 1111 Catherine St, Ann Arbor, MI 48109 USA

**Keywords:** Female genital mutilation, Female genital cutting, Prevalence, Factors, Ghana

## Abstract

**Background:**

Globally, three million girls are at risk of female genital mutilation (FGM) and an estimated 200 million girls and women in the world have undergone FGM. While the overall prevalence of FGM in Ghana is 4%, studies have shown that the overall prevalence in the Upper East Region is 38%, with Bawku municipality recording the highest at 82%.

**Methods:**

This study used a cross-sectional design with a quantitative approach: a survey with women of reproductive age (15–49).

**Results:**

Among all respondents, 830 women who participated in the study, 61% reported having undergone FGM. Of those circumcised, 66% indicated their mothers influenced it. Three quarters of the women think FGM could be stopped through health education. Women who live in the Pusiga district (AOR: 1.66; 95% CI: 1.16–2.38), are aged 35–49 (AOR: 4.24; 95% CI: 2.62–6.85), and have no formal education (AOR: 2.78; 95% CI: 1.43–5.43) or primary education (AOR: 2.10; 95% CI: 1.03–4.31) were more likely to be circumcised relative to those who reside in Bawku Municipal, are aged 15–24, and had tertiary education. Likewise, married women (AOR: 3.82; 95% CI: 2.53–5.76) were more likely to have been circumcised compared with unmarried women. At a site-specific level, factors associated with FGM included age and marital status in Bawku, and age, marital status, and women’s education in Pusiga.

**Conclusion:**

Female Genital Mutilation is still being practiced in the Bawku Municipality and the Pusiga District of northern Ghana, particularly among women with low socio-economic status. Implementing interventions that would provide health education to communities and promote girl-child education beyond the primary level could help end the practice.

## Background

Female genital mutilation (FGM), also known as female circumcision or female genital cutting, is defined as the partial or total removal of external female genitalia and injury to the female organs for cultural or other nontherapeutic reason [1]. FGM is performed in various forms in 28 African countries, and the social drivers behind the practice are multifaceted. Globally, three million girls are at risk of genital mutilation, [2] and an estimated 200 million girls and women in the world have undergone FGM [1].

Although the overall prevalence of FGM in Ghana is 4% [3], studies have shown that the prevalence varies by region and is widespread in northern Ghana [4–6]. In the Upper East region, clinical research revealed an overall prevalence of 38%, with Bawku municipality recording the highest at 82% [7]. It is hypothesized that the higher prevalence of FGM in Northern Ghana resulted from the mixture of the people and culture of Northern Ghana with those of the neighboring countries of Mali, Togo and Burkina Faso, where the practice is more common.

Women and girls who have undergone FGM are at risk of both short- and long-term consequences. The short-term risks of FGM include severe pain, excessive bleeding, shock, genital tissue swelling, heightened risk of human immunodeficiency virus (HIV) after using the same knife to cut women/girls [8], impaired wound healing, psychological consequences, and even death. Long-term consequences of the practice include infections, such as chronic genital, reproductive tract, and urinary tract infections, and pain, including painful urination, painful intercourse and menstrual problems [8, 9]. Other risks of FGM include keloids, obstetric complications, perinatal risks, reduction of sexual quality, dyspareunia, and psychological consequences among other things [2, 10–12].

Several studies have examined factors that influence the practice of FGM. Some have highlighted important relationships between demographic factors such as age, education and religion [12–15]. Studies in sub-Saharan African countries suggest a relationship between economic factors and female circumcision [13, 14]. Women with better financial resources or household affluence were less likely to be circumcised [13–15], being younger and better educated has been shown to be protective of FGM [13–16].

Cultural and religious factors have been found to influence the practice of FGM [13, 16–20]. Cultures that put a high premium on preservation of virginity, reducing premarital sex and early pregnancy, and minimizing the risk of extramarital affairs have been shown to be more likely to encourage FGM [16, 21, 22].

### Justification

Many women have undergone female genital mutilation in Ghana and many more women and girls are at risk of undergoing female genital mutilation every year. The majority of girls are cut before they turn 15 years old. In 1994, the Ghanaian government outlawed female circumcision. According to this law, circumcisers can be sentenced to up to 3 years of imprisonment (Criminal code Amendment Bill; Ghana, 1994) [23]. However, this traditional practice is still going on among some predominant ethnic groups of the Upper East region in spite of the 1994 legislation against it.

Moreover, clinical studies have revealed that FGM is detrimental to reproductive health [5, 24–30], but the practice is reported to be sustained by traditional and social values [4, 31] that need to be understood and addressed by intervention programs.

The aim of the study was to determine circumcision status of women of reproductive age and factors associated with it in the Bawku Municipality and Pusiga District of northern Ghana.

## Methods

### Study design and methods and setting

This study used a cross-sectional design with a quantitative approach: a survey of women of reproductive age (15–49 years) to determine the prevalence and determinants of FGM.

The study was conducted in the Bawku Municipal and Pusiga District. Bawku is one of the thirteen [12] districts and municipalities in the Upper East Region of Ghana. It shares boundaries with Burkina Faso, the Republic of Togo, Bawku West District and Garu-Tempane District to the north, east, west and the south respectively. The administrative capital is Bawku. The population of the district according to 2010 Population and Housing Census stands at 98,538 with 47,254 males and 51,284 females [32].

**Fig. 1:**
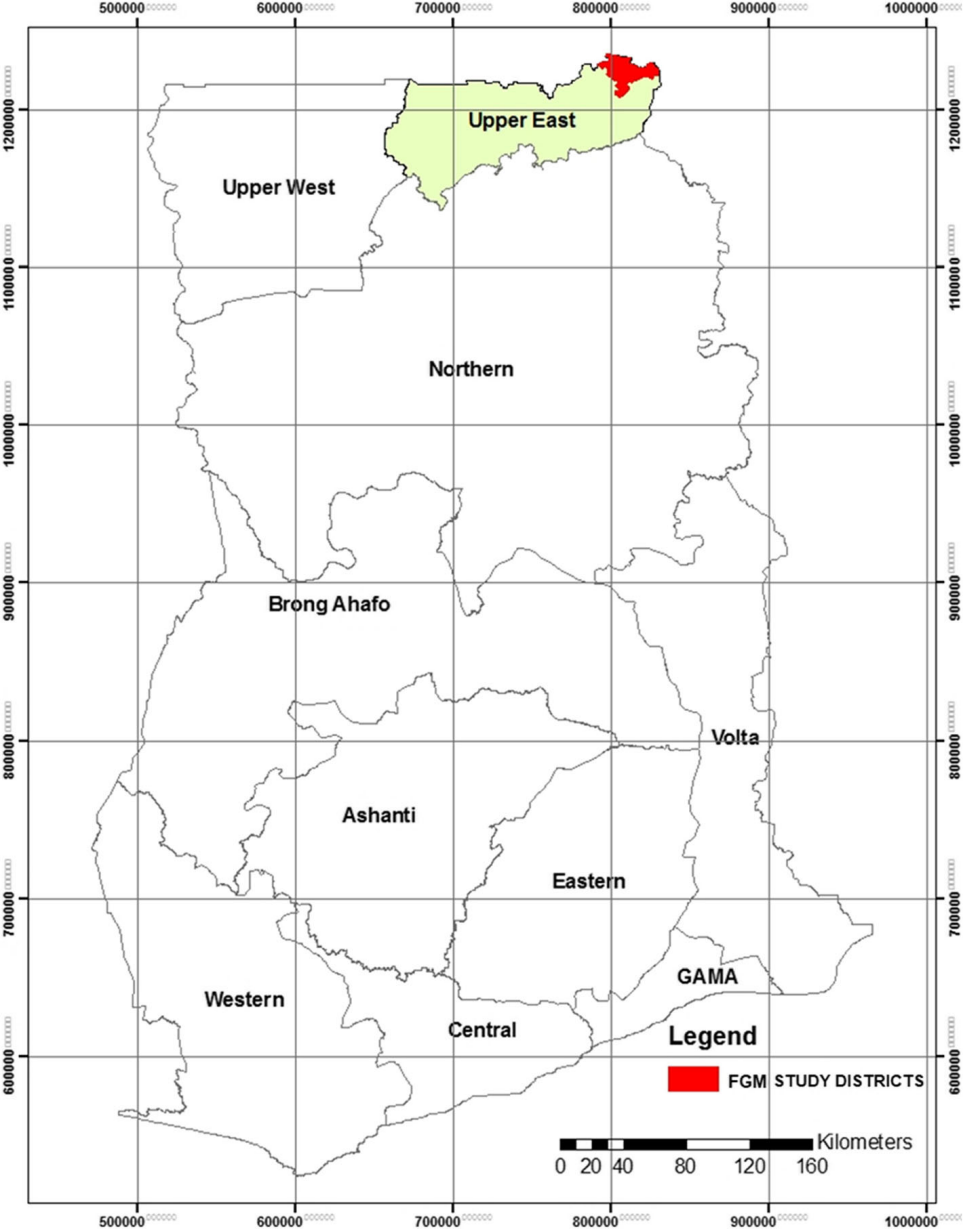
Source: Map of Ghana showing the geographical location of the study sites. http://www2m.biglobe.ne.jp/ZenTech/world/map/Ghana/Outline_Map_of_Ghana.htm

Pusiga District with its administrative capital Pusiga is one of the 13 administrative and political districts in the Upper East Region of Ghana and was carved from Bawku Municipal in 2012. Pusiga shares boundaries with Burkina Faso to the North, Republic of Togo to the East, Bawku West to the West and Bawku Municipal to the South. The population of the district according to 2010 Population and Housing Census stands at 57,677 [32] (Fig. 1).

### Sampling and participant criteria

The sample size was calculated based on the population of women aged 15 and 49 years (Bawku = 24,494, Pusiga 15,040, total population = 43,038) and an average estimated proportion of FGM (50%) in the Bawku Municipality and Pusiga District with a 95% confidence interval as well as a corresponding *p* < 0.05 for significance. We used the formula for sample size *n* = {2(*Z*a + *Z*1–*β*) ^2σ2,^ / Δ2} [33] and this gave a sample size of 379 women in Bawku Municipality and 375 women in Pusiga District. We assumed a refusal rate of 10%, meaning one would need to interview approximately 417 women in the Bawku Municipality and 415 women in the Pusiga District. The total sample size for the two districts was 832 women.

A two-stage sampling method was used and the primary sampling unit is the community. The first stage involved selecting 8 communities in the Bawku Municipality and Pusiga District. In the second stage, a random direction from the center of the community was selected, by spinning a pen. The houses along that direction were counted out to the boundary of the community, and one selected at random was the first household surveyed. Proximity selection was used to select subsequent households as the “next nearest” until the desired sample size was reached. We repeated that in all the selected communities until we attained the number of respondents required. The selection of the number of households to be interviewed in each community was based on the size of the population of the community. We interviewed one woman within the reproductive age (15–49 years) in each household. Where there was more than one eligible respondent, we randomly selected and interviewed one person. For the random selection process, each eligible member of the household was assigned a unique number, then each number placed in a bowl and mixed thoroughly. The fieldworker then randomly picked numbered tags from the bowl and interviewed the selected women. We included 830 women in the analysis and excluded two because of missing key background information. The inclusion criteria of participants was all women of reproductive age (15–49 years old) who reside in the selected communities of the two study sites.

We collected the data using a structured questionnaire that included variables about social and demographic characteristics such as age, education, religion, father’s religion, mother’s religion, marital status, ethnicity, geographical location (i.e. Bawku and Pusiga), and household assets. These independent variables were collected based on previous studies [12–16, 34, 35]. We generated a quintile rank for wealth, based on possession of 23 items representing household assets.

The questionnaire was developed in the English language and pre-tested outside the study communities in order to improve the relevance and appropriateness of the questions. The pretest offered the fieldworkers the opportunity to practice the interviewing techniques, and the questionnaire was revised appropriately after the exercise. The fieldworkers underwent 2 weeks of training prior to the survey and visited households to interview eligible women.

### Data analysis

We produced descriptive statistics to summarize respondents’ background characteristics. Multivariable logistic regression analysis was performed to identify factors associated with circumcision status among women of reproductive age in the two study sites. We determined whether the respondents had been circumcised by asking the question, “Are you circumcised”? The following explanatory variables were analyzed: geographical location, age, marital status, educational level, religion, ethnicity, wealth index, father’s education and mother’s education. The wealth index consisted of 23 household-related items. We generated quintile ranks for wealth status using principal component analysis. All *P* values were two-tailed, and the significance level was set at *p* < 0.05. We performed all statistical analyses using Stata Version 12 (Stata Corp., TX).

## Results

### Respondents’ socio-demographic characteristics

Table 1 shows the characteristics of the 830 participants from the two sites included in the study. The number of respondents from each of the district is about the same – 417 in the Bawku Municipality and 413 in Pusiga. Approximately 57% of the women had received no education. Sixty seven percent of the women were aged 15 to 34 years. The majority (82%) of the women were Moslems and 8% identified themselves with the Christian faith.

**Table 1:** Socio-demographic characteristics of respondents

Characteristics	Bawku Municipal (*n* = 415) N (%)		Pusiga District (*n* = 415) N (%)		Both Districts (*n* = 830) N (%)	
Age group						
15–24	158	38.07	139	33.49	297	35.78
25–34	126	30.36	136	32.77	262	31.57
35–49	131	31.57	140	33.74	271	32.65
Religion						
Traditional	3	0.72	1	0.24	4	0.48
Christianity	37	8.92	55	13.25	92	11.08
Islam	375	90.36	359	86.51	734	88.44
Father’s Religion						
Traditional	30	7.23	40	9.64	70	8.43
Christianity	21	5.06	61	14.70	82	9.88
Islam	364	87.71	314	75.66	678	81.69
Mother’s Religion						
Traditional	21	5.06	26	6.26	47	5.66
Christianity	36	8.67	72	17.35	108	13.01
Islam	358	86.27	327	76.39	675	81.33
Ethnicity						
Kusassi	80	19.28	97	23.37	177	1.33
Mamprusi	5	1.20	6	1.45	11	21.33
Busanga	302	72.73	204	49.16	506	60.96
Moshie	25	6.02	44	10.60	69	8.31
Hausa	1	0.24	10	2.41	11	1.33
Other	4	0.48	54	13.01	56	6.74
Marital status						
Married	285	68.67	298	71.81	583	70.24
Single/widowed/separated	130	31.33	117	28.19	247	29.76
Education						
None	246	59.28	225	54.22	471	56.75
Primary	57	13.73	83	20.00	140	16.87
Middle/JSS/JHS	90	21.69	65	15.66	155	18.67
Secondary/SSS/SHS +	22	5.30	42	10.12	65	7.71
Wealth Index						
Poor	104	25.62	169	41.01	273	33.37
Middle	135	33.25	138	33.50	273	33.37
vRich	167	41.13	105	25.49	272	33.26
Circumcision Status						
Yes	236	56.87	273	65.78	509	61.33
No	179	43.13	142	34.22	321	38.67
Most Important Reason for Practice						
It’s necessary puberty rites	9	2.17	9	2.17	18	2.17
Control sexual desire	145	34.94	96	23.13	241	29.04
Continue a tradition	137	33.01	227	54.70	364	43.86
For social acceptance	100	24.10	65	15.66	165	19.88
Other	24	5.78	18	4.34	42	5.05
How to Stop FGM						
Education	312	75.19	318	76.63	630	75.90
Prosecute practitioners	97	23.37	90	21.69	187	22.53
Alternative puberty rites	4	0.96	4	0.96	8	0.97
Other	2	0.48	3	0.72	5	0.60

In addition, 61% of them were of the Busanga tribe and 70% were married. In all, 61% of women reported having undergone FGM and of those circumcised, 66% indicated their mothers influenced it. And the most important reasons for the practice included to continue a tradition (44%), control sexual desire (29%), and for social acceptance (20%). About three quarters of the women think FGM could be stopped through health education.

### Factors associated with circumcision status across study sites

Table 2 presents the results of a regression analysis for circumcision status by selected characteristics across the two sites. The results revealed that women who reside in the Pusiga district (adjusted odds ratios [AOR]: 1.66; 95% CI: 1.16–2.38), are aged 35–49 (AOR: 4.24; 95% CI: 2.62–6.85), and have no education or primary education (AOR: 2.78; 95% CI: 1.43–5.43) (AOR: 2.10; 95% CI: 1.03–4.31) were more likely to circumcise relative to those who reside in the Bawku Municipal, are aged 15–24 and had tertiary education respectively. Also, married women (AOR: 3.82; 95% CI: 2.53–5.76) were more likely to have been circumcised compared with the unmarried (i.e. single, divorced, or widowed).

**Table 2:** Regression Analysis Results for Circumcision Status Across the two Study Districts

Characteristic	OR	(95% CI)	AOR	(95% Cl)
Geographical Location				
Bawku (r)	**1**		**1**	
Pusiga	**1.46**	1.10–1.93**	**1.66**	1.16–2.38**
Age-group				
15–24 (r)	**1**		**1**	
25–34	**2.60**	1.85–3.66***	1.24	0.82–1.88
35–49	**9.84**	6.50–14.91***	**4.24**	2.62–6.85***
Religion				
Other religions (r)	**1**		**1**	
Islam	1.33	0.87–2.04	1.45	0.73–2.91
Father’s Religion				
Other religions (r)	**1**		**1**	
Islam	**0.34**	0.18–0.63***	0.96	0.33–1.73
Mother’s Religion				
Other religions (r)				
Islam	0.82	0.57–1.18	0.99	0.43–2.27
Ethnicity				
Kusassi (r)	**1**			
Busanga	1.09	0.77–1.55	1.16	0.73–1.83
Moshie	1.02	0.58–1.80	1.18	0.58–2.38
Other	0.85	0.49–1.45	0.77	0.39–1.54
Woman’s education				
None	**6.81**	3.85–12.02***	**2.78**	1.43–5.43**
Primary	**3.40**	1.81–6.37***	**2.10**	1.03–4.31*
Middle/JHS	0.99	0.53–1.85	1.26	0.62–2.58
Secondary/SSS/SHS+ (r)	**1**		**1**	
Marital Status				
Divorced/widowed/never married (r)	**1**		**1**	
Married	**6.68**	4.81–9.28***	**3.82**	2.53–5.76***
Wealth index				
Poor (r)	**1**		**1**	
Middle	1.12	0.80–1.59	0.98	0.64–1.48
Rich	**1.42**	1.01–2.01* 1.21		0.80–1.85

Bold values are significant (*p < 0.05; **p < 0.01; ***p < 0.001). *AOR* adjusted odds ratio, *CI* confidence interval, *JHS* junior high school, *JSS* junior secondary school, *O.R* odds ratio, *SHS* senior high school, *SSS*: senior secondary school

### Factors associated with circumcision status at each study site

Table 3 presents the results of the regression analysis for circumcision status according to selected characteristics, in the Bawku Municipal. The results revealed that, women aged 35–49 (AOR: 5.05; 95% CI: 2.48–10.30) and were married (AOR: 4.83; 95% CI: 2.61–8.93) were more likely to circumcise relative to 15–24 years old and the unmarried respectively.

**Table 3:** Regression Analysis Results for Circumcision Status in the Bawku Municipal

Characteristic	OR	(95% CI)	AOR	(95% Cl)
Age group				
15–24 (r)	**1**		**1**	
25–34	**1.84**	1.14–2.95**	0.92	0.51–1.58
35–49	**10.8**	5.98–19.6***	**5.05**	2.48–10.30***
Religion				
Christianity (r)	**1**		**1**	
Islam	1.52	0.79–2.92	0.93	0.28–3.11
Father’s Religion				
Other religion (r)	**1**		**1**	
Islam	0.48	0.21–1.12	2.02	0.45–9.05
Mother’s Religion				
Christianity (r)	**1**		**1**	
Islam	0.12	0.64–1.97	0.63	0.15–2.72
Ethnicity				
Kusassi (r)	**1**		**1**	
Mamprusi	1.03	0.63–1.69	1.10	0.55–2.23
Busanga	0.99	0.40–2.46	1.11	0.36–3.42
Moshie	1.30	0.30–5.80	3.71	0.65–21.1
Woman’s education				
None	**6.57**	2.47–17.5***	1.12	0.62–7.28
Primary	**3.18**	1.09–9.30*	1.97	0.52–7.34
Middle/JSS/JHS	0.97	0.34–2.77	0.95	0.26–3.53
Secondary/SSS/SHS+	**1**		1	
Marital Status				
Divorced/widowed/never married (r)	**1**		**1**	
Married	**7.71**	4.80–12.4***	**4.83**	2.61–8.93***
Wealth index				
Poor (r)	**1**		**1**	
Middle	1.03	0.62–1.71	0.97	0.51–1.81
Rich	1.60	0.98–2.65	1.78	0.96–3.29

Bold values are significant (*p < 0.05; **p < 0.01; ***p < 0.001).*AOR* adjusted odds ratio, *CI* confidence interval, *JHS* junior high school, *JSS* junior secondary school, *O.R* odds ratio, *SHS* senior high school, *SSS* senior secondary school

Table 4 presents the results of a regression analysis for circumcision status by selected characteristics across in the Pusiga District. The results revealed that, women aged 25–34 (AOR: 1.97; 95% CI: 1.08–3.59) and 35–49 (AOR: 4.27; 95% CI: 2.18–8.33), who had no education (AOR: 3.20; 95% CI: 1.40–7.31) and were married (AOR: 2.84; 95% CI: 1.59–5.10) were more likely to be circumcised compare with their counterparts, aged 15–24, unmarried and had tertiary education respectively.

**Table 4:** Regression Analysis Results for Circumcision Status in the Pusiga District

Characteristic	OR	(95% CI)	AOR	(95% Cl)
Age group				
15–24 (r)	**1**		**1**	
25–34	**3.69**	2.23–6.10***	**1.97**	1.08–3.59*
35–49	**8.89**	4.96–15.9***	**4.27**	2.18–8.33***
Religion				
Other religions (r)	**1**		**1**	
Islam	1.29	0.72–2.30	1.74	0.72–4.18
Father’s Religion				
Other religions (r)	**1**		**1**	
Islam	**0.24**	0.09–0.66**	0.51	0.18–1.42
Mother’s Religion				
Other religions (r)	**1**		**1**	
Islam	0.71	0.43–1.17	1.20	0.42–3.38
Ethnicity				
Kusassi (r)	**1**		**1**	
Mamprusi	1.35	0.81–2.26	1.29	0.68–2.45
Busanga	0.99	0.47–2.07	1.21	0.48–3.04
Moshie	0.71	0.38–1.33	0.66	0.30–1.44
Woman’s education				
None	**8.22**	4.00–16.9***	**3.20**	1.40–7.31**
Primary	**3.72**	1.71–8.16***	2.02	0.84–4.87
Middle/JSS/JHS	1.17	0.52–2.64	1.22	0.49–3.02
Secondary/SSS/SHS + (r)	**1**		**1**	
Marital Status				
Divorced/widowed/never married (r)	**1**		1	
Married	**5.82**	3.66–9.25***	**2.84**	1.59–5.10***
Wealth index				
Poor (r)	**1**		**1**	
Middle	1.37	0.85–2.20	1.09	0.61–1.93
Rich	1.46	0.87–2.45	0.84	0.45–1.55

Bold values are significant (*p < 0.05; **p < 0.01; ***p < 0.001). *AOR* adjusted odds ratio, *CI* confidence interval, *JHS* junior high school, *JSS* junior secondary school, *O.R* odds ratio, *SHS* senior high school, *SSS* senior secondary school

## Discussion

The results revealed that 61% of women in the Bawku Municipality and the Pusiga District have been circumcised. Factors associated with FGM in the two sites included the woman age, marital status, women’s education and geographical location. At a site-specific level, the following factors were associated with FGM: age and marital status in Bawku and age, marital status and women’s education in Pusiga.

Geographical location played a significant role in women circumcision status: Women/girls in the Pusiga District had a higher probability of being circumcised than their counterparts in the Bawku District. The reason could be that more women in Pusiga might have been crossing the border to neighboring Togo to circumcise since the practice is outlawed in Ghana [24]. Moreover, Bawku unlike Pusiga is a Municipality with more development programs [34, 36], making it more likely for women to receive health education and information on the harmful effects of the practice for informed decision making on whether or not to be circumcised. Besides, Bawku compared with Pusiga has achieved some improvement in post-secondary and tertiary education [34, 36] and that might have contributed to a reduction in FGM, as highly educated women are more likely to oppose the practice [17, 35, 37, 38]. Other studies also showed significant geographic variations of FGM within and across countries.

The results also revealed that women’s age is a strong determinant of circumcision. The women aged 34–49 years old were more likely to be circumcised compared with their younger counterparts. The reason could be that in the past, almost all women in the Bawku Municipality and the Pusiga District embraced female circumcision, making it hard or impossible to come across a woman who had not undergone the practice [7]. However in recent times, FGM had slightly declined among the youth possibly because of human rights and legal protection of women and girls against the practice [23]. FGM is now carried out in secrecy [39] in some communities or not at all in others because of the 1994 law that seeks to sentence any circumciser and other perpetrators up to 3 years imprisonment [23]. Such penalties could have contributed to the reduction in the number of circumcised women and girls yearly. These findings corroborate results by Setegn et al. [14], Bogale et al. [40] and Rahlenbeck and Mekonnen [41] that showed higher age categories were associated with increased odds of FGM among women .

Married women were more likely to have undergone FGM compared with their unmarried counterparts across the two study sites. Female genital mutilation is typically seen as a rite of passage into womanhood and a precursor to marriage [22, 42]. Once marriage is crucial to women/girls and FGM enhances their marriageability, they are forced to be mutilated [43]. The role of marriage in perpetuating the practice is understood in the following ways: FGM helps to preserve virginity, controls girls’ and women sexuality [44–47] and is seen as a prerequisite for marriage [44, 45, 48–50]. Many studies have identified marriage as a factor associated with FGM [4, 46, 47, 51, 52].

We found that women with tertiary education were less likely to be circumcised compared with their counterparts with no education or primary education, particularly in the Pusiga district. Research has shown that educated women are more likely to weigh the benefits over the risks before making decisions about their health [43]. A clear understanding of the benefits of not circumcising is likely to be attractive to women. In addition, when women are able to make autonomous decisions, they are able to take actions that will favor them [53–55]. Some studies report that educated women wield economic power and thus have the authority to make independent decisions about FGM [3, 35, 56, 57]. Efforts towards boosting women’s empowerment, including deliberate policies to ensure girl-child education beyond the primary level, have huge potential in ending female circumcision. Previous studies corroborate the finding that higher education achieved by women and girls could reduce female circumcision [17, 35, 37].

## Limitations

This study has a number of limitations. First, recall bias could have limited the validity of the data, because some participants could have forgotten about past events involving FGM. Using different local languages to collect the data could also have distorted the presentation of the questions to the respondents. However, the standard training for fieldworkers and supervisors and the in-depth translation and back translation of the questions minimized the language bias.

## Conclusion

Despite a decline in female circumcision among women of reproductive age in the Bawku Municipality and the Pusiga District, FGM is still practiced. Implementing an intervention targeting community members, particularly women with little education, and intensifying girl-child education in those settings might have a huge impact on eradicating the practice.

## Abbreviation

FGM: Female Genital Mutilation
